# Older Adults with Hoarding Behaviour Aging in Place: Looking to a Collaborative Community-Based Planning Approach for Solutions

**DOI:** 10.1155/2012/205425

**Published:** 2011-10-12

**Authors:** Kyle Y. Whitfield, Jason S. Daniels, Keri Flesaker, Doneka Simmons

**Affiliations:** ^1^Faculty of Extension, University of Alberta, Enterprise Square, Room 2-215, 10230 Jasper Avenue, Edmonton, Alberta, Canada T5J 4P6; ^2^Department of Educational Psychology, University of Alberta, 6-102 Education North, Edmonton, Alberta, Canada T6G 2G5; ^3^Senior Association of Greater Edmonton, 15 Sir Winston Churchill Square, Edmonton, Alberta, Canada T5J 2E5

## Abstract

This paper reports on and synthesizes new research that examines how a collaborative community response can promote successful aging in place for older adults with hoarding behaviour. Through interviews with older adults with hoarding behaviour, who used a particular community support and a focus group interview with members of the community collaborative that directed supports for this population, our findings suggest that there were valuable outcomes for both groups. These older adults with hoarding behaviour were able to remain in their own homes, their safety was enhanced, their sense of isolation was minimized, empowerment was fostered, and they gained valuable insight into their behaviour. The members of the community collaborative were able to access the expertise of other professionals, maximize their own expertise, and they generated an enhanced understanding of the experience of older adults living with hoarding behaviour in Edmonton. This study is a significant addition to the much too sparse literature about the community planning needs of older adults with hoarding behaviour. It offers knowledge that is integral to theories and principles of better aging in place but attempts to translate this into practice.

## 1. Introduction

Older adults with hoarding behaviour are often at a high risk of being homeless making aging in place extremely complex. This paper reports on a study that examines the value of a community-based planning approach that responds to the needs of this population, a population that is both increasing in number and that is very seldom studied [1]. It synthesizes new research about the complexities associated with remaining in one's own home when he/she is over 55 and has compulsive hoarding behaviour. And it examines how a collaborative community response promotes successful aging in place for this population. Not only are community-based services necessary to better understand because they are central to all health sectors [2], but also current research surrounding those with hoarding behaviour is mostly focussed on methods addressing individual-level behavioural characteristics of hoarding through cognitive behavioural therapy (cf., the extensive work of Frost and Steketee). Missing are descriptions of community-based planning approaches for health and social service sectors working hard to make aging in place a possibility.

For older individuals with hoarding behaviour, aging in place is complex because hoarding behaviour is multifaceted; it touches on social, environmental, familial, and personal issues [3]. Aging in place is also not simple for those with hoarding behavior because they want to remain living in their own homes, neighbourhoods and communities which seem to require that a collection of agencies, often representing different sectors, understand their needs in order to help them stay in the community and age in place. In this paper, aging in place refers to an ideal where people can age in the familiarity of their homes, neighbourhoods, and communities where their quality of life is maximized by the availability and accessibility of supports and services that respond to their needs and capacities [4, 5]. Aging in place, in addition, is about belonging to a community that supports one's many needs, for example, physically, socially, mentally, environmentally, and so forth [6]. To understand the varying aspects of aging in place in late life, older individuals with hoarding behaviour need to be further understood so their aging journey is successful; collaborative approaches by community support agencies can help to make that possible. 

In our case, social and health related organizations from different sectors, that in some way supported people with hoarding behaviour in Edmonton, AB, Canada, were brought together in 2007 through the leadership of the social worker of a seniors support agency called SAGE (Seniors Association of Greater Edmonton). After having visited a number of individuals with hoarding behaviour (age 55+) in her professional role, she noticed, as did those members of the imminent community collaborative, that those with hoarding behaviour were at a high risk for being evicted from their homes, and they experienced shame associated with their hoarding resulting in isolation, as well as depression; they were at risk of falling in their own homes, and generally, they were living in unsanitary conditions. To approach this highly vulnerable population, to respond to some of their needs, especially to prevent their potential eviction, a concerted effort by a broad representation of social, health, and other agencies was necessary. The collaborative met together regularly (e.g., once every 2 months over the years and as of June 2011, it continues to meet) to continually plan for and improve the support of older adults with hoarding behaviour in the Edmonton area. 

Although little is still known about the effects of one's neighbourhood on the mental health of older adults, positive mental health in later life may be influenced by the way in which older people feel about their neighbourhood [7]. It is fair to conclude, therefore, that for older adults with hoarding behaviour, place matters and the role of place as locality is key when making meaning at both the individual and the collective levels. In particular, local place is an important factor in identity, in ones sense of community, and attachment; this is crucial to the determinants of attachment, satisfaction, and behaviour [8]. Also important is place and the person-place relationship are very much grounded in a context of fear over loss of place [9] and the obliteration of locality [10]. Aging and compulsive hoarding behaviour need to be examined in concert with one another. 

As our aging population rapidly increases, the number of older adults with hoarding behaviour will also quickly increase. Although hoarding behaviour usually begins in early adolescents, its severity increases with age [11]. Results from the Hopkins Epidemiology of Personality Disorder Study found that the odds of hoarding were over two times as great in the oldest compared with the youngest age group [12], and in a study of hoarding-related complaints to public health departments in Massachusetts, 40% of individuals that hoarded were involved with elder service agencies [13]. 

Referring to Frost and Hartl [14] when defining hoarding behaviour, there are key characteristics: the acquisition of and failure to discard a large number of possessions that seem to be of little use or limited value; cluttered living spaces that cannot be used as intended; significant distress and/or impairment associated with the clutter. Hoarded possessions clutter living spaces until rooms are difficult or impossible to use for their designed purpose, causing significant stress to the individuals themselves. Possessions may be purchased through compulsive buying [15] and/or through acquiring things like newspapers or discarded items from dumpsters [16]. Some individuals may even steal new possessions. After acquiring possessions, discarding these items is extremely difficult [17]. In serious instances, household clutter may interfere with and prevent daily activities, like food preparation, moving freely in one's home, and using the bathroom [11, 17]. And as clutter increases, falling, fire, sanitation issues, depression, and isolation are possible, creating significant risk for a number of outcomes but especially eviction and thus homelessness [18].

Community collaboration is one way to respond to social and health needs of a population. In fact, genuine collaborate is say to be the very thing that successfully reforms health systems [14]. Although there are many ways to define collaboration, most definitions emphasize the importance of shared responsibility and a team approach [20], and using a collaborative approach can significantly increase the available pool of resources from which team members can draw [21]. A response to the multiple challenges of older adults with hoarding behaviour requires a comprehensive and far-reaching approach, more than one single agency can provide alone [22, 23]. Philosophically, collaboration is rooted in systems theory which says that entities in a system are dependent on one another [24] and ecological theory in particular proposes that causes and solutions of health and/or social problems are beyond the individual and are associated with such determinants as the health and social services that we receive [25]. Service recipients can benefit from a collaborative approach to the provision of community health services as can the agencies participating in the collaborative [26–28]. Using a collective made up of representative agencies to support this vulnerable population creates potential for an approach that builds on the strengths of all those involved.

## 2. Methods

Between January 2007 and January 2010, approximately 75 older adults (ages 55+) with hoarding behaviour in Edmonton, AB, Canada, were provided with community supports to prevent them from being evicted from their homes [29]. The Seniors Association of Greater Edmonton (SAGE) offers support through a program referred to as *This Full House*. And *This Full House* is a direct outcome of the work of the community collaborative. The aim of *This Full House* for older individuals with hoarding behaviour is to prevent eviction from their home, improve their health and well-being, maintain positive social contacts, and contribute to the building of a healthy community [29]. As the population of potential participants with hoarding behaviour in Edmonton is not particularly large, a small-n approach was used in an attempt to create heuristic generalizations which Tsoukas [30] defines as opportunities to refine analytic understanding and to make more incisive distinctions than were previously possible. Small-n studies are not designed to support or refute a theory, but rather, to further refine it. As such, the purpose of this study is to further examine the role of a collaborative planning approach in a community setting when seeking to help those over 55 years with issues relevant to having compulsive hoarding behaviour and wanting to age well in one's community. 

To further understand the value of collaboration and, in particular, its role and value in this community support, interviews and a focus group were conducted with seniors with hoarding issues involved with *This Full House *and with the community collaborative. All interviews were semistructured and conducted by a third party researcher (i.e., Research Assistant). Ethics approval for this research was received from the University of Alberta, Research Ethics Board.

### 2.1. Interviews with Individuals with Hoarding Behaviour

Individual interviews were conducted with five (*N* = 5) individuals with hoarding behaviour involved with *This Full House*, all of whom were over the age of 55. The semistructured, face to face interviews took place at a location expressed as being most comfortable to the interviewee, for example, at their homes, at a university office, at a nearby coffee shop. These study participants were first contacted by the social worker from SAGE who directs *This Full House*. The individuals with hoarding behaviour had either referred themselves to *This Full House* or had been referred to it by one of their health care practitioners or family members. At an appointment with the individuals, the social worker informed them about the study asking if they might be interested in being interviewed by a researcher about their experience with *This Full House.* If they agreed (which all five did), she gave them a one-page written description of the study. She then went through the summary of the study ensuring they understood what was being requested from them. With their agreement, their telephone numbers were provided to the researcher (i.e., Research Assistant) who contacted them and established a location and time to meet for the interview. The aim of the one-hour interviews was to gather information regarding their experience with *This Full House*. In particular, the interview was used to understand their perceptions of the impact of or value of their association with *This Full House*, that is, the aspects of the program they benefitted from most.

### 2.2. Focus Group with the Community Collaborative Members

A community collaborative made up of social and health related agency representatives providing insights into the ongoing development of *This Full House* was formed. The focus group interview included ten members (*N* = 10) (of the possible 11 members in total) and was conducted in a face-to-face manner for approximately 1.5 hours. Members of this collaborative represented a number of expert groups: social workers, home care nurses, geriatric neuropsychologists, geriatric nurses, fire and safety investigators, public health practitioners, and environmental health and safety officers. The focus group questions centered on the nature of the working relationship between and amongst the members and their observations about the value and impact their work may have had on the service users, that is, those with hoarding behaviour. The questions guiding the semistructured interview were generated from key themes highlighted in the literature (i.e., health services, collaboration, community support, etc.) that aligned with the purpose of the study.

## 3. Results

Use of a grounded theory framework was quite valuable for our data analysis. Grounded theory means that the data analysis essentially, is “grounded” in the data [31]. Therefore, the concepts and themes we describe in our results have evolved from and are embedded in the data collected and have been mined through a process of conceptual ordering. Grounded theories are said to provide further insight, to enhance understanding, and to be used as a guide to inform action (i.e., acting on the results). As Strauss and Corbin [31] describe in more detail, our interview data, in the form of pages of the exact words from the interview, was organized into categories that were not predetermined but that evolved after reading and rereading this data many times. And that, mainly described ideas and offered explanations from interviewees that had commonalities to each other. The themes described here are those that were mentioned frequently and carried the same meaning. 

Because our aim in this study was to further our understanding of how older adults with hoarding behaviour were supported by a particular community-based planning approach, grounded theory provided the most effective means of organizing, reducing, and understanding the data. Suggested below is a picture that describes how the work of the community collaborative, because of its high level of collaboration, resulted in many important benefits for older people with hoarding behaviour that align well with and facilitate the goals and ideals associated with an aging in place model. The picture also describes how the community collaborative members valued their experience on the team.

### 3.1. The Work of the Community Collaborative Benefitted People with Hoarding Behaviour

As a result of this group working together to respond to the needs of older adults with hoarding behaviour, several themes evolved from the data demonstrating direct benefits for these individuals: being able to remain in their own homes; reducing their potential for harm, and minimizing their isolation all which allowed them to experience a feeling of empowerment which also helped them to generate insight into issues surrounding their hoarding behaviour.

#### 3.1.1. Remaining in Own Home

There are considerable challenges associated with aging in one place for older individuals with hoarding behaviour. They can be at significant risk of being evicted from their homes and their behaviour can be a major public health concern leading to eviction as a result of violating building, fire, or property maintenance codes. It may not be until a particular emergency occurs (i.e., water leakage, fire, and pest infestation) that a landlord is notified [32]. One focus group member describes their role, as a member of the community collaborative, in minimizing evictions for this population:

*“Our legislation says that we do have the right to go into any public or private place if we believe there may be a public health nuisance… and if that means we have to order their suite cleaned out, we'll do it. Um, because you can't get control of bedbugs and cockroaches unless you treat all the suites and if somebody's hoarding, you can't get rid of them… so, so they have to clean up.”*

The five interviewees in this study were all in a situation where eviction from their homes was a potential, but they were able to remain at home as a result of a community-based approach that addressed some of their needs. Prior to the existence of this community collective, one focus group member describes how the health inspector had to play all roles and visit clients once a week and “nag people into cleaning up, which was mostly unsuccessful.” For example, when there is a potential home eviction for individuals with hoarding behaviour, the social worker and a public health worker, together with the client and other members of the collaborative, that is, where necessary such as a fire and safety representative, provide input into the problem-solving process. A professional organizer usually assists with the practical aspects associated with cleaning up including heavy lifting, removing garbage, and reorganizing resulting in “[these clients being able to] stay living at home without being on the street and [being] homeless.” Being able to age in one's own home, one's neighbourhood, and community fosters independence significantly impacts a more positive relationship in the person-place relationship [33].

The five people with hoarding behaviour that we interviewed spoke about the value of being able to remain living in their own homes. One person found it motivating to have someone help him/her to clean up his/her apartment, “it's the motivation of having someone there plus… the helper doing the heavy lifting and heavy carrying… making… 76 thousand trips to the garbage bin… its stressful but helpful [and] I certainly would not have been able to hire a company on my own […] if the program had not been in effect [and] I would be in deep do-do with Capital Health.” For another individual, what helped him remain in his own home was having the home care worker put him in touch with the social worker and the cleaning person who helped him find ways to deal with issues of parting with his stuff. And for this gentleman, the social worker supported him by suggesting options: “she made life more convenient for me by offering me options.” And for another person, the social worker and the cleaner reminded her that the condition of her house, the bugs, the mouse droppings, the make up from years ago, the shiny covered magazines all over, and the infestation that she had, was not her fault and she could probably face organizing and cleaning it with encouragement and the help of a plan.

#### 3.1.2. Reducing Harm and Promoting Safety

Hoarding behaviour creates a significant safety risk for the individual him/her self and for the community [32]. Harm reduction is a core principle that is essential to address the needs of those with compulsive hoarding behaviour because promoting safety is foremost [13]. In this case, focusing on harm reduction by the community collective ensured that safety was embedded within all the actions, initiatives, and supports they provided. One focus group member talks about the value of taking on this strategic focus: “We subsequently learned the value of focusing on a harm reduction approach wherein we address issues of harm first so that the person [with hoarding behaviour], at least, will be safe. Even though they may be living with a significant amount of stuff every day of their lives, but at least they are safe.” A harm reduction philosophy considers behaviour change to be incremental and assumes that people will maintain their behaviour change when they have decision-making power to influence their goals and put them into action [34]. 

This focus on safety and reducing harm or the potential for harm helped the individuals with hoarding behaviour buy into the larger process at hand, that is, to contribute to the building of healthy neighbourhoods, supporting their well-being, and helping them stay in their homes as long as possible. Instead of suddenly or immediately removing the person from a potentially unsafe environment or situation, the aim instead is first to reduce the potential for harm and create a safe place to live. In the case of these five individuals, it meant such things as: hiring a person to help them remove and reorganize their excessive items, getting help to fumigate their apartments, openly talking about their hoarding situation to help them reflect upon it, receiving nonjudgemental support, and establishing a plan to minimize household items. One interviewee describes the value of setting goals and generating a plan, “we set a plan and the social worker would come back and generally we would accomplish that goal whatever it was.” It also meant helping the person at risk of potential eviction, for example, to respond to requests made by the Public Health Inspector. One person with hoarding behaviour describes the role of this service (i.e., *This Full House*): “they sort of mediate [between varying agencies] and rub off the sharp corners.” Reducing harm by promoting safety enables an aging in place philosophy and model as both can facilitate positive and long-term aging in one location.

#### 3.1.3. Minimizing Loneliness

The community collaborative, used in this study as a planning approach helped to address the problem of isolation for these older adults. Several of the representative organizations of the community collaborative, that is, a social worker, public health nurse, geriatric nurse, and so forth, offer home visitation to many of their clients and observe their living environment. As observed by one community collaborative member, “a lot of these seniors are very lonely, very isolated, and so the fact that they have someone that's coming to their home often, helps.” Another member talked about the impact of the support group (which is provided for people with hoarding behaviour on a monthly basis as part of *This Full House* services) on minimizing their sense of loneliness and connecting with people that have similar experiences: “[there is] value of that coming together, meeting with other people and seeing and hearing that you're not alone.” Two interviewees with hoarding behaviour confirmed this same sentiment, about the importance of feeling connected to a group: “the group has helped me” and “[I realized] you are not the odd one out.” As a result of not feeling alone and part of a group, interviewees with hoarding behaviour said that they felt empowered.

#### 3.1.4. Fostering Empowerment

Empowerment can be an outcome of collaborative relationships; it offers a catalyst for new community programs and other supports, changes in policies, and advancing health practices [35, 36]. The members of the community collaborative intentionally aimed to facilitate empowerment using it as a principle to guide their work that addressed the needs of older individuals with hoarding behaviour in the greater Edmonton area. During the focus group interview, one member describes how empowerment as a guiding principle was translated into action benefitting a particular individual with hoarding behaviour:

*“We [the community collaborative] have entertained some really creative approaches in terms of dealing with management [i.e., housing manager] and having the client lead those interventions […] as opposed to, we [the service providers] meet[ing] with management, then… meet[ing] with the client… we really include the client in those interventions, so that the client really is aware of everyone that's involved, what's being discussed and then they are really empowered to be part of the action plan.”*

When individuals with hoarding behaviour are more involved in directing their own support, they may experience greater control in such decision making which can lead to empowerment [13]. As observed by another community collaborative member, “[empowerment provides] a sense of control in a situation that they may feel a lack of control.”

#### 3.1.5. Communicating Insight into Their Hoarding Behaviour

The most successful reported treatment for those with compulsive hoarding behaviour is the use of behavioural treatment, in other words, a cognitive behavioural model [37]. To be motivated to discard their possessions, insight into their hoarding behaviour is significant [14, 38]. Members of the community collaborative said that individuals with hoarding behaviour seemed to gain insight into their behaviour as a result of the support provided by this collaborative. This observation was expressed during the focus group interview by the social worker:

*“… the insights that come out as a part of the intervention, as you go along, then they [clients with hoarding behaviour] start to reveal some insight as to “how did I ever get to this place? and, “I can't believe this happened to me” and “I can't believe that I'm actually, I'm making some decisions now that I was not able to before”.”*

During an individual interview, one individual with hoarding behaviour reflected on the changes in her own behaviour of accumulating things: “I would still be walking down one little path between the bathroom, bedroom and one side of the kitchen and that would have been it.” Another interviewee with hoarding behavior expresses insight into her hoarding behavior: “they [the social worker] finally were able to get me to accept… this condition… that it probably was not my fault and that I could probably face it… it's been a great support… eye opening.” And another person said something similar: “when I think how far I came from the first day…. it [my behavior] improved.” She also said “I need to continue both thinking about and maybe following up […] with counseling […] is there an answer why I have become a person who allows clutter around myself […]?”. In the one-on-one interviews, individuals reported an improved feeling of independence and a sense of empowerment. This occurred because the collaborative team joined forces, they were united by a common goal of supporting, to the best of their ability, the needs of this population who they noticed to be struggling more and more and who they were being called upon more frequently to try to assist.

### 3.2. Participating in the Community Collaborative Benefitted the Group Members, Individually and Collectively

On individual and collective levels, the members of the community collaborative experienced three significant benefits as a consequence of participating in this group. For example, members of the collaborative were able to access the expertise of other professionals, they maximized the use of their own skills and knowledge and significantly enhanced their understanding of hoarding behaviour.

#### 3.2.1. Accessing Expertise from the Other Group Members

Working on the collaborative team allowed the individual members an opportunity to access a broader range of skills and knowledge than those who were found solely in their own area of expertise or their own organization. In one instance, a professional social worker described how she could now present the risks associated with hoarding behaviour more objectively to a client with greater confidence as she could make reference to and more easily call on the authoritative role of the local fire department. Because the firefighter and social worker were both members of the collaborative, a close working partnership was facilitated. This benefit was expressed in this way: “when I mention to her [the client with compulsive hoarding behavior] the possibility of having someone from [the fire department] come and just do an assessment to let her know what her risk level is [i.e., of eviction from her home], she was suddenly open to that.” As a result of working collaboratively, members also got to know more about the professional resources available to them in the community through their ongoing communication together. As one health professional of the community collaborative said: “it is a professional benefit to see and use the expertise around this table for the benefit of the individual clients.” 

Evaluating collaborative planning practices must consider not just the purpose of the collaboration but the value of its relational interactions [28]. For example, asking how social relations are changed can reveal how certain conditions are impacted for the group. Access to new areas of expertise is one descriptor of the quality of social relations. The sharing of expertise between and amongst the members was said to directly benefit the older people with hoarding behaviour using the services of *This Full House*. In the words of one member of the community collaborative, “a benefit of the collaborative process was working with everybody, to partner, to ensure that we're getting our clients the best support.”

#### 3.2.2. Maximizing their Own Expertise

Participating in this group allowed team members to maximize the use of their own expertise. One member, a public health inspector, recounted a time when such a collaborative approach was not used to support older adults with hoarding behaviour illustrating the tremendous limitations of working in isolation:

*“Before… [the community collaborative existed] it was [up to]… the health inspector to try and play all roles and just sort of go and visit once a week and try and nag people into cleaning up. Which was mostly unsuccessful and wasn't really our job. I mean we are not social workers, we're not mental health workers, we are public health inspectors.”*

Functioning alone, the health inspector had to operate as the only contact for this client group. Working in isolation took away time from the job he/she was actually trained and hired to do forcing them to work beyond their professional scope of practice. As further evidence of the value of being able to maximize one's own expertise, the professional organizer, who provides hands-on assistance with the cleanup of client homes, can now maximize her cleaning and organizational skills while directing clients' emotional issues to a trained professional. As described by the social worker during the focus group interview: “A big portion of her [the professional organizer's] time was addressing the [clients] emotional issues. So we've now learned that when those issues come up, it's a direct link back to me.”

#### 3.2.3. Enhancing Their Knowledge of Hoarding Behaviour

Members of the community collaborative described how their partnering with one another as professionals helped to enhance their knowledge and understanding of compulsive hoarding behaviour. Gaining new knowledge and a more “enlightened understanding” of compulsive hoarding behaviour was said to be the result of participating in this ongoing process. One member of the collaborative described the value of the increase of his knowledge stating “another professional benefit [of being a member of the community collaborative] is deepening my personal understanding of what hoarding is and what the dynamics are. Certainly, it's helped me in recognizing that it's multi-faceted.” Group members said they were then able to take their learning back to their representative organizations: “I think working with [the collaborative] has really helped to educate me and hopefully the rest of [name of organization].” Successful aging in place requires that support by community-based organizations exists that it is available and accessible and responsive to a variety of their needs; therefore, knowledge about aging and its long list of associated issues, such as compulsive hoarding behaviour, is imperative.

## 4. Discussion

The aim of our discussion is to explain several matters that underpin the major themes of our results. Explaining why such themes evolved and their relationship to the broader phenomenon being studied is what Strauss and Corbin refer to as the process of theorizing [31]. At the heart of this study is community-based planning as a phenomenon of which a number of related concepts are embedded: aging in place, social support, collaboration, vulnerable populations, and community services. Overall, our research attempted to discover how a collaborative approach to planning for and addressing the needs of older adults with hoarding behaviour, living in the community, provided value. For these vulnerable adults living in Edmonton, Alberta, Canada, a collaborative planning approach that involved multiple agencies (representing varying sectors) that worked continuously to improve their quality of life made a difference. And, the members of this community collaborative also benefitted. From this approach they were able to access the professional expertise of the other group members, maximize the use of their own skills and knowledge, overall, giving them an opportunity to generate new insights into hoarding behaviour which they described as helping them provide the best possible care and support to this population. It is feasible; therefore, to use such results to inform the many ways to age in place more successfully in late life. 

Currently there is no systemic, long-term process to support older people living in the community that have compulsive hoarding behavior. Nor in Canada, is there an overall strategy to plan for our aging population, therefore aging in place, at a national, political scale, is not yet a priority. But, because people with this behavior will increase in numbers, and the complexities associated with their need to live safely in their own communities, a national strategy must also address their specific and unique needs. As emphasized by The Canadian Health Services Research Foundation [39], our study also finds a need for enhanced integration, cooperation, and coordination at the system and at the service delivery levels. That is, integration and collaboration between health and social services, between ranges of sectors, between disciplines of front line workers, and between government ministries. Collaboration and integration need to be part of the foundation upon which aging in one's own home and community can be realized. In addition, our study supports the findings of Keonig et al. [3] who found that when having to facilitate ethical dilemmas for this population, older adults with hoarding behavior benefit from the use of teams whose members have a variety of disciplines. 

Well evidenced in the health services planning literature, applied to an aging population, is the need for improved collaboration, both at a principle and a practice-based level [35]. Our study also confirms and emphasizes this important need. Not only do our findings align with the literature but it provides further insight into the challenges associated with older individuals that want and deserve to remain in their own homes. Our particular case is specific to older individuals that have hoarding behaviour that were supported through the efforts of a community collaborative planning approach. Our study adds to the current aging in place literature and extends it. The current literature supports collaboration and integration at varying levels of the health and social support system. But studies that examine community planning approaches grounded in collaboration are uncommon. This population will increase in numbers over time and they deserve to remain in their own communities with the support of surrounding agencies and organizations that work togather to best support older individuals with hoarding behaviour to age well in their own homes.

## 5. Conclusions

At the heart of several concepts and themes arising from interviews with older people with compulsive hoarding behavior and members of a community collaborative working to support this population is an approach founded on collaboration between and amongst service providers. Results demonstrated that when a highly collaborative approach to planning is used, there were quite direct benefits for older adults with hoarding behavior and, at the same time, there were benefits for the members of the community collaborative. This approach to planning for the health and social needs of this population resulted in people with hoarding behavior being able to remain in their own homes when eviction was a potential, enhancing their safety, helping to minimize their isolation, and creating opportunities to increase control in their own decision making. The members of the community collaborative could now access the expertise of other professionals, maximize their own expertise, and they generated new insight and understanding of the experience of older adults living with hoarding behaviour in Edmonton. Our study needs to be viewed within certain boundaries. Although our data conveys that this approach to planning has quite positive outcomes, our data is short term and situational. Our use of a single, one-time only interview method only allows us to draw insights and observations about that moment in time and not over an extended trajectory. As well, our study is grounded in five interviews with older adults with compulsive hoarding behavior. And although a collaborative approach to addressing the needs of older people with hoarding behaviour conveyed comprehensive benefits, collaboration as a planning approach is rarely the complete answer or solution to people's social and health needs. Health Integrated Delivery systems, for example, are far more comprehensive but do view collaborative planning as a core principle [2]. Viewed in this light, aging in place may not always be possible, but it must be realized that community-level social and health related supports maximize the quality of later life while aging at home [5]. And further building on that is the need for a well-coordinated model of care [5, 35] where supports are comprehensive, easily accessible, and well connected [5].
